# A Case of Superficial Femoral Arteriovenous Fistula and Severe Venous Stasis Ulceration, Managed with an Iliac Extender Prosthesis

**DOI:** 10.1155/2017/9460958

**Published:** 2017-07-20

**Authors:** Nicole Ilonzo, Selena Goss, Chun Yang, Michael Dudkiewicz

**Affiliations:** Mount Sinai St. Luke's-West, New York, NY, USA

## Abstract

Most femoral artery arteriovenous fistulas occur as a result of percutaneous interventions. However, arteriovenous fistulas can occur in the setting of trauma, with resultant consequences such as heart failure, steal syndrome, or venous insufficiency. Indications for endovascular repair in this setting are limited to patients who are at too high risk for anesthesia, have a hostile groin, or would not survive significant bleeding. We report the case of a traumatic femoral arteriovenous fistula, causing severe venous insufficiency and arteriomegaly, in a 58-year-old male, with history of traumatic gunshot wound complicated by popliteal DVT. Surgical options for arteriovenous fistula include open and endovascular repair but this patient's fistula was more suitable for endovascular repair for reasons that will be discussed.

## 1. Introduction

This is a case of an arteriovenous fistula causing severe venous insufficiency with ensuant venous stasis ulceration. Venous insufficiency is a disease that affects anywhere from <1 to 40% of females and <1 to 17% of males [1]. Patients with chronic venous insufficiency can develop complications from this disease, with the most severe being venous stasis ulcerations.

## 2. Case Presentation

**Figure 1:**
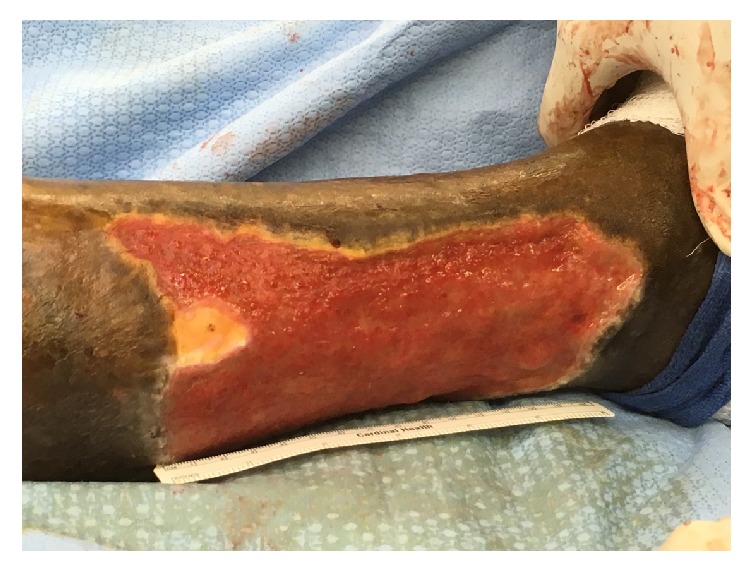
Right lateral leg venous stasis ulcer.

**Figure 2:**
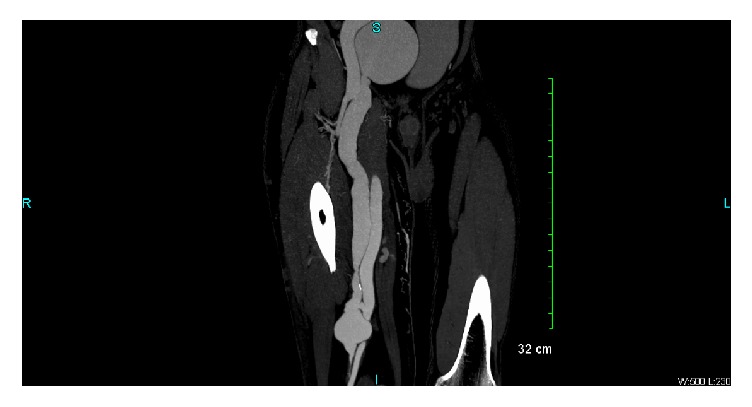
CT angiogram showing a superficial femoral arteriovenous fistula with arteriomegaly of the right external iliac artery.

A 58-year-old male presented to the emergency department with right lower extremity swelling and a large ulceration on his right lateral leg for nearly six years (Figure 1). The patient had been treated in Guyana with topical agents and dressing changes, without improvement. In the ED, right lower extremity duplex demonstrated popliteal vein thrombosis as well as traumatic fistula between superficial femoral vein and superficial femoral artery. Duplex ultrasound also showed distal femoral vein to measure 4.36 cm AP, mid femoral vein 1.12 cm, and an enlarged lymph node noted in the groin measuring 4.56 × 1.60 cm. CT angiogram of the abdomen, pelvis, and lower extremities showed right superior femoral arteriovenous fistula and a tortuous right common iliac artery that may be causing a May Thurner's type compression of the R common iliac vein (Figure 2).

**Figure 3:**
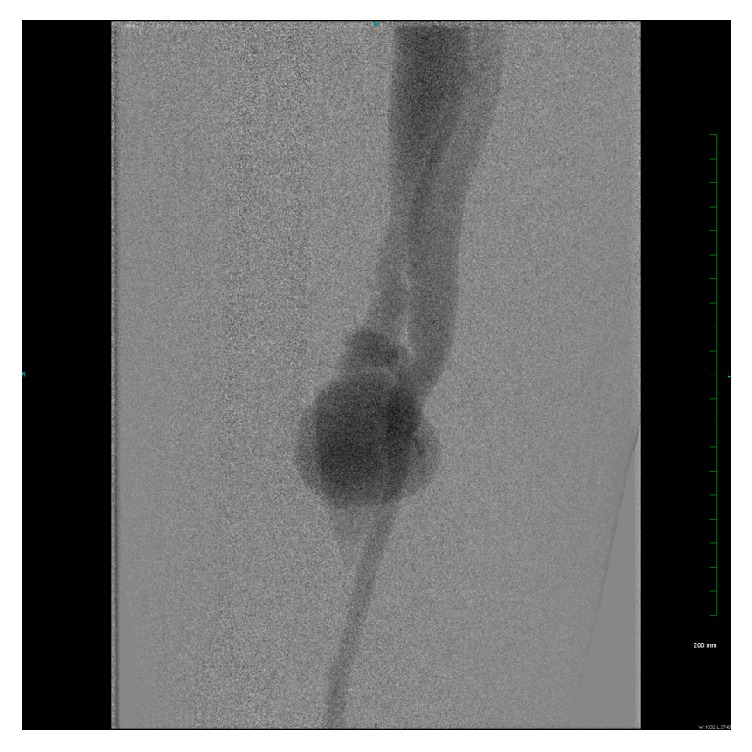
Right lower extremity angiogram showing a patent and enlarged right superficial femoral artery with a distal AV fistula connecting the superficial femoral artery to the femoral vein with venous flow upwards.

**Figure 4:**
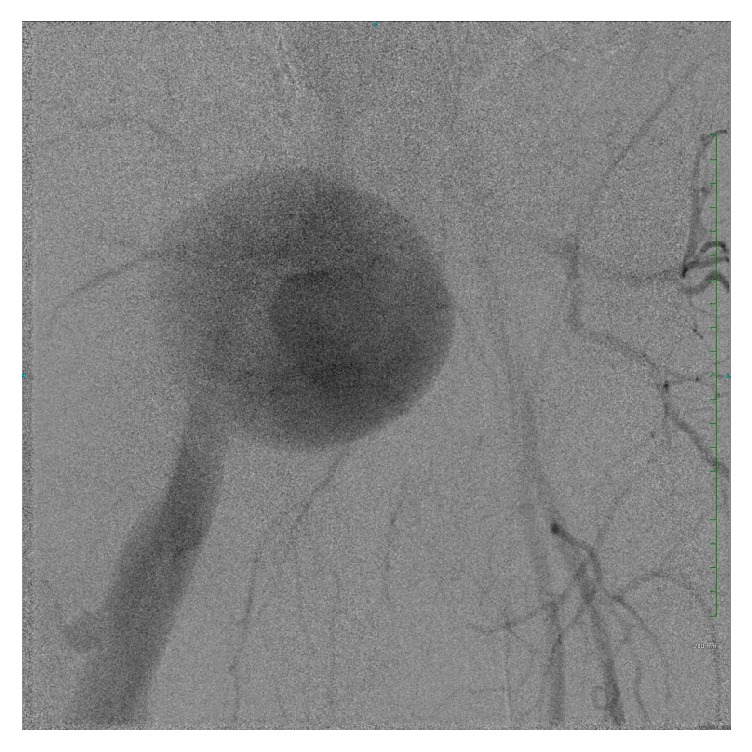
Right lower extremity angiogram showing possibility of a venous malformation, which was not seen on previous imaging.

Patient subsequently underwent bilateral extremity angiogram and selective catheterization of the right common iliac artery via retrograde left femoral approach. Angiogram revealed a patent but enlarged right common femoral artery, a patent right profunda artery, and a patent and enlarged right superficial femoral artery (SFA) with a distal arteriovenous (AV) fistula connecting the superficial femoral artery to the femoral vein with venous flow upwards (Figure 3). On pelvic imaging, there also appeared to be very late venous filling possibly of a venous malformation, which was not seen on previous imaging (Figure 4). There was a large venous aneurysm just distal to the fistulous tract. The remainder of the arterial outflow was normal giving rise to a patent popliteal artery and patent tibial trifurcation.

**Figure 5:**
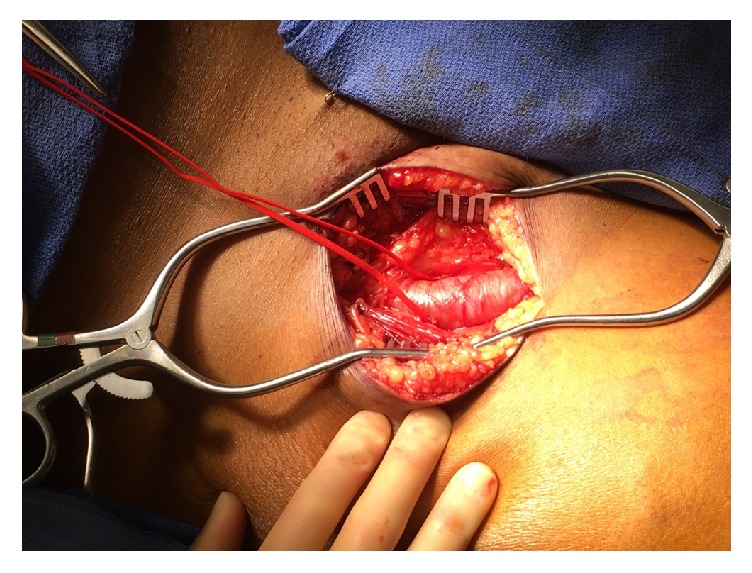
A superficial femoral artery cutdown was performed revealing an enlarged SFA.

**Figure 6:**
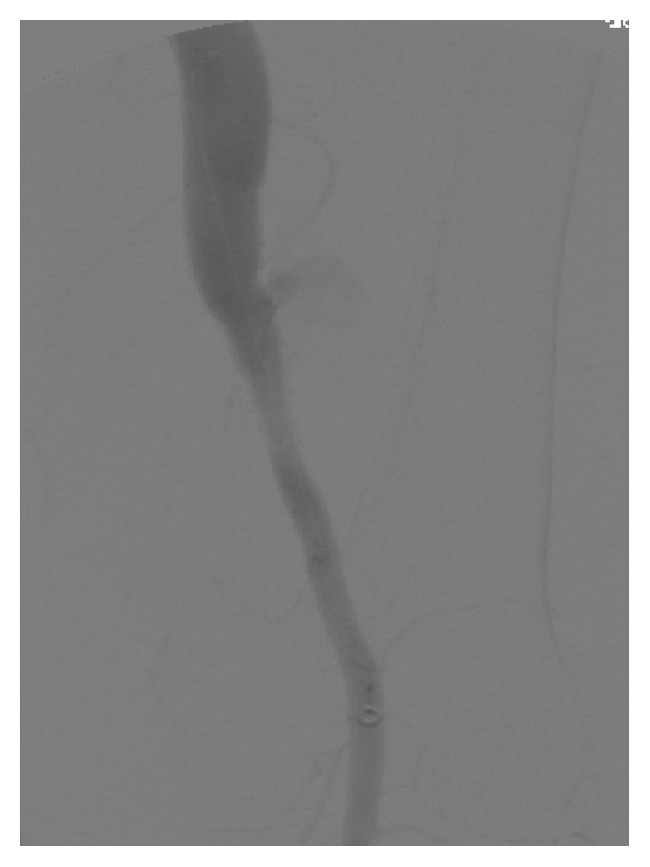
Superficial femoral arteriovenous fistula occluded after placement of iliac extender stent.

**Figure 7:**
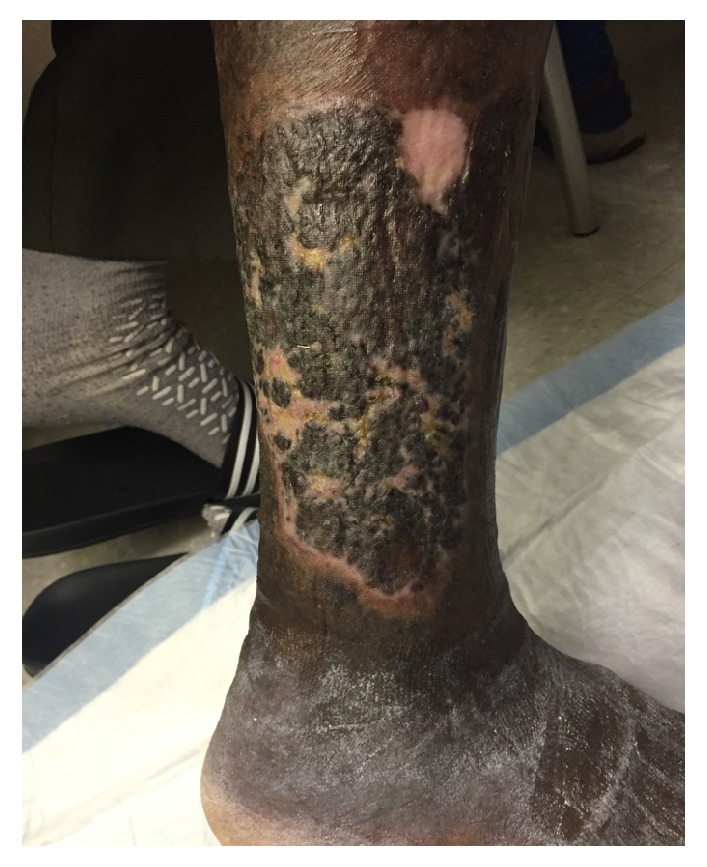
Wound healed within 3 months.

The decision was made to pursue endovascular repair of the patient's fistula. Given concerns that the SFA was so enlarged and at risk for significant hemorrhage, a superficial femoral artery cutdown was performed (Figure 5). The SFA was dissected free from its surrounding structures. The anterior wall of the SFA was punctured in an antegrade fashion and a 12-French sheath was then placed. There was some bleeding around the 12-French sheath which required clamping of the proximal superficial femoral artery with a vascular clamp. A Gore iliac branch endoprosthesis which measured 10 mm distally 16 mm proximally for a length of 7 cm was opened and deployed in standard fashion across the arteriovenous fistula. It was postdilated using an angioplasty balloon. After postdilation angioplasty there was good apposition against the wall and the fistula filled very slowly via collaterals and not via inline flow (Figure 6). In addition, the patient maintained his outflow via the popliteal artery. Split thickness graft was placed over the ulcer and negative pressure therapy was utilized. The VAC was taken down and there was excellent take of the skin graft. Patient has subsequently undergone Unna booth therapy and the wound has healed completely after 3 months (Figure 7).

## 3. Discussion

The scoring system used to classify and stage venous insufficiency is called the clinical manifestations, etiological factors, anatomical distribution, and pathophysiological conditions classification (CEAP classification) [2, 3]. This patient, with a CEAP classification of C6 for active venous ulceration, required surgical intervention. The key to treatment of this venous stasis ulceration was to interrupt the fistula [4].

Traumatic arteriovenous fistulae can be repaired by open or endovascular approaches [5, 6]. This patient's fistula was best managed with an endovascular approach but there were limitations given the proximal and distal landing zones were significantly different in size. Using a standard Viabahn stent would be a suboptimal choice for this reason. Therefore, the decision was made to use a Gore Excluder Iliac branch endoprosthesis, which could adjust for this asymmetry. As described by the manufacturer, the Gore Excluder Iliac branch endoprosthesis is indicated for treatment of infrarenal artery aneurysms. The graft is exclusively designed for the iliac artery [7]. However, it was used with excellent effect in this context.

Of note, there may be an element of residual venous hypertension secondary to compression of the right external iliac vein by the hypertrophied right external iliac artery. We anticipate further healing of his venous stasis ulcer now that the patient's venous insufficiency is largely corrected with the stent. However, if his venous disease did not seem to improve significantly, we would have attempted to treat the external iliac vein compression with a stent in hopes of providing patency to the vein.
